# The Design of RFID Convey or Belt Gate Systems Using an Antenna Control Unit

**DOI:** 10.3390/s110909033

**Published:** 2011-09-21

**Authors:** Chong Ryol Park, Seung Joon Lee, Ki Hwan Eom

**Affiliations:** Department of Electronic Engineering, Dongguk University, 3-26, Pil-dong, Joong-gu, Seoul, Korea; E-Mails: parkcr@dongguk.edu (C.R.P.); acousticjoon@naver.com (S.J.L.)

**Keywords:** RFID tag, RFID conveyor belt gate system, antenna control unit, recognition rate

## Abstract

This paper proposes an efficient management system utilizing a Radio Frequency Identification (RFID) antenna control unit which is moving along with the path of boxes of materials on the conveyor belt by manipulating a motor. The proposed antenna control unit, which is driven by a motor and is located on top of the gate, has an array structure of two antennas with parallel connection. The array structure helps improve the directivity of antenna beam pattern and the readable RFID distance due to its configuration. In the experiments, as the control unit follows moving materials, the reading time has been improved by almost three-fold compared to an RFID system employing conventional fixed antennas. The proposed system also has a recognition rate of over 99% without additional antennas for detecting the sides of a box of materials. The recognition rate meets the conditions recommended by the Electronic Product Code glbal network (EPC)global for commercializing the system, with three antennas at a 20 dBm power of reader and a conveyor belt speed of 3.17 m/s. This will enable a host of new RFID conveyor belt gate systems with increased performance.

## 1. Introduction

Radio Frequency Identification (RFID) is the technology that recognizes moving physical objects and transmits information about the materials to a server through certain network environment to manage the data of many materials. Recently, the range of applications of RFID is gradually expanding to many areas such as access, asset and supply chain management. The general RFID system is comprised of RFID tags, readers, antennas and middleware. The tag has a unique code for each material, recognized by a Radio Frequency (RF) signal from the reader, and transfers code information to the reader. Then the RFID reader transmits the code information received from the RFID tags to a DB (Database) through middleware. Compared to the barcode systems used in asset management the most outstanding features of the RFID system are reading distance, durability, and simultaneous recognition ability [1].

**Figure 1.**
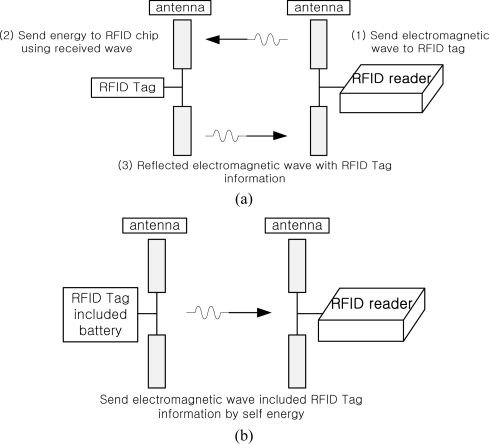
Operation of two types of RFID tag. (**a**) Passive type RFID tag; (**b**) Active type RFID tag.

RFID tags can be divided into two types according to the power supply method: active type or passive type, as shown in Figure 1. An active type tag can generate its own power which can improve the RFID reading performance, but a passive one cannot produce electric power itself.

The two types of RFID tag are applied by a variety of methods according to the various environments in the field of material distribution management. The passive tags, in general, are more used in supply chain management due to their cheaper price.

**Figure 2.**
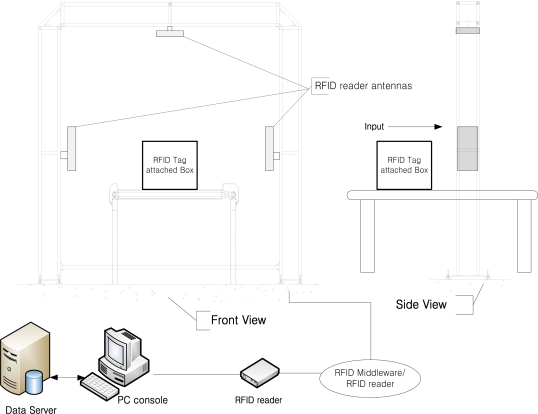
Standard structure of RFID conveyor belt system.

As plenty of materials are coming in and out of warehouses during a certain period of time, more precise and prompt checking of materials is required to manage a lot of materials. For this reason, in order to realize real-time verification system of great quantity of materials at the gate of RFID system, conveyor systems are used at the warehouse sites. Various methods such as inserting an antenna under the conveyor belt, employing numerous antennas on the gate or using directional antennas are used to realize a conveyor belt system combined with a RFID system for real-time management of materials [2–4]. The final aim of these methods is to increase the recognition rate of the RFID tags when the system is applied in commercial sites. Figure 2 shows the standard structure of a conveyor system, and the system can be applied to diverse areas.

In this paper, we propose the design of a RFID conveyor belt gate system using an antenna control unit for efficient material management. The proposed antenna control unit, driven by a motor and located on top of the gate, has an array structure of two antennas with parallel connection to improve the directivity of the antenna beam pattern and the RFID reading distance. The Electronic Product Code (EPC) global network (EPCglobal) has proposed an experimental method with specific standards in order that the RFID system of the conveyor belt system can be used anywhere in the world [4]. Therefore, experiments in this paper were carried out according to the method recommended by EPCglobal. The effectiveness of the proposed system was verified and compared with conventional a conveyor belt gate system by diverse experiments. The recognition rate of the proposed system which has only two antennas attached on the antenna control unit meets the conditions recommended by EPCglobal.

The rest of this paper is organized as follows: the experimental method and conditions proposed by EPCglobal, and examples and the performance of common conveyor systems are described in Section 2. In Section 3, the design concept of the proposed antenna control unit with novel configuration and the operation of the entire system is represented. Section 4 gives some experimental results of our RFID conveyor belt gate system employing antenna control unit. The conveyor belt system was running at a speed of 1, 1.5, 2, 2.5 and 3.2 m/s (the recommended velocity condition of EPC global is 3.17 m/s). Some concluding remarks were given in Section 5.

## 2. Conventional RFID Portal Systems

EPCglobal, founded in 2003, is a nonprofit organization that provides standardized product management systems, system commercialization and code management, and also promotes related business globally. The RFID systems obtained from the EPCglobal network are used to manage precise distribution of innumerable materials at industrial sites. RFID technology and its application methods are being continuously developed in various fields. Wal-Mart, airport logistics and container management are representative cases of successful distribution management using RFID conveyor belt gate systems which are the most popular method for realizing real-time supply chain management. The conveyor belt system can be applied to materials as diverse as liquids, metals, and paper boxes [5–8].

The most significant objectives of material distribution using RFID conveyor belt systems are precise and prompt management of mass materials. In order to achieve these, high recognition rates are required when materials move at a high speed on a conveyor belt system, and the RFID system should also follow the standards proposed by EPCglobal, which recommends the following experimental conditions; the speed of the conveyor belt is 190.5 m/min (3.17 m/s), the distance between portal gate and conveyor belt is 965 mm, RF power from the reader is 10–20 dBm, and a circularly polarized wave antenna is used as a reader antenna. To determine the starting point where a reader recognizes RFID tags, a sensor is used as a detector. With this standardized experiment, the effectiveness of any RFID conveyor belt gate system can be demonstrated in actual sites.

**Figure 3.**
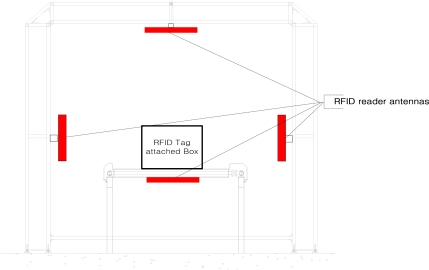
The RFID conveyor belt gate structure proposed by EPC global.

The experimental setup proposed by EPCglobal (see Figure 3) has four antennas located on four faces of the gate. This method can be evaluated with the assumption that RFID tags are attached on all faces of a box passing through conveyor system. The experimental conditions of the proposed conveyor belt gate system with antenna control unit based on the experimental standards of EPCglobal will be discussed in Section 3.

## 3. Design of the Antenna Control Unit

The antenna control unit has two patch antennas arranged in parallel on the reader. This structure has the same effect of array antennas but a longer recognition distance. As discussed in several papers and analysis [9–12], by conversion between two antennas which both have the same antenna gain on the array antenna, the beam pattern of the array antenna is sharpened, and the antenna gain is improved without output variation, so in this paper, in order to improve gain and readable range, an array structure of two patch antennas was connected to antenna control unit.

**Figure 4.**
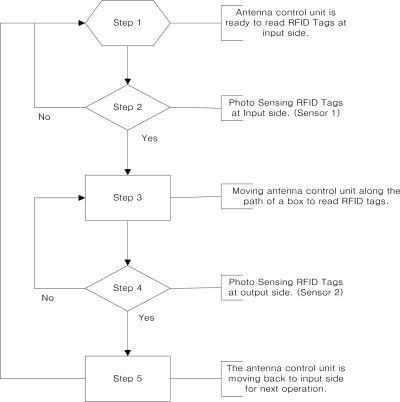
Operation flow of antenna control unit.

To make the reading time of the reader longer to increase the recognition rate, the antenna control unit is placed immediately along the path of materials with a constant angular velocity which is the same as speed of conveyor belt when a box is detected by a photo-sensor located in front, 1.5 m away from the gate. The operation of the antenna control unit is finished when the materials pass by a photo-sensor located at the back, 1.5 m away from the gate. The antenna control unit is designed in that the direction of an antenna can be immediately returned onto starting position when the reading operation is ended. The returning velocity of the antenna control unit is the same as the speed of the conveyor belt. We assume that each material on the conveyor belt is spaced at regular intervals following the velocity of the conveyor belt. Figure 4 shows the operation flow of the antenna control unit.

**Figure 5.**
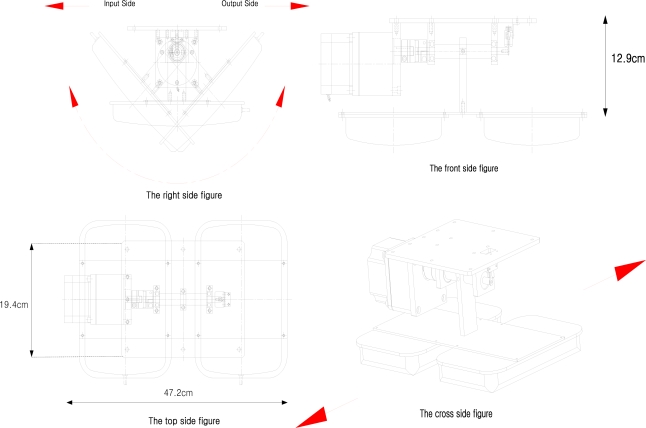
A drawing of the antenna control unit.

Since the antenna control unit is designed to extend the readable distance by arraying two antennas, it rotates with an operating range of 45° for both the front and back sides of the gate for effective tag identification. This can help to prevent unnecessary reading action of the antenna control unit for effective recognition of materials within gate boundaries. Figure 5 shows drawings of the designed antenna control unit.

The antenna control unit is attached at the top side of the gate and is controlled at the same speed of conveyor belt. In general, the recognition rate of RFID systems is influenced by power, reading time and the directivity of the RFID reader antenna, performance of the tag antenna, and the speed of conveyor belt [12–15]. We could also verify the relationship between the reading time and the speed of the conveyor belt in this paper. In the case where a fixed RFID antenna is used, the reading time is reduced by increasing the speed of the conveyor belt. When the antenna control unit moves with a constant angular velocity which is the same as the speed of conveyor belt used for the system, the reading time increases more about three times compared to the case of the fixed type antenna.

In a RFID conveyor belt gate system which must check the quantity of materials, the speed of the conveyor belt can seriously affect effective material management. The conveyor belt speed recommended by EPCglobal is 3.17 m/s and nearly 100% recognition rate is required for commercializing a system. Therefore, the antenna control unit of the proposed system has a velocity range of up to 3.2 m/s which can satisfy the recommendations of EPCglobal for commercialization.

## 4. Implementation and Experimental Results

Following the standards of EPCglobal, experiments with the antenna control unit were carried out to validate the recognition rate of RFID tags attached to a box while passing it through the conveyor belt gate system. The experimental results with the antenna control unit were compared with the ones obtained with a fixed antenna structure, and with three antennas attached on three faces of the gate. From these experiments, we can see that the reading time has certain relationships with the location of attached tags, the polarization characteristics of the reader antenna and the speed of the conveyor belt.

### 4.1. Fixed Antenna & Proposed Antenna

**Figure 6.**
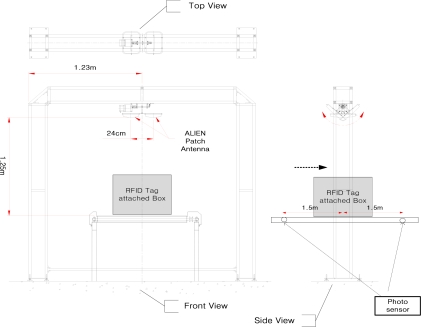
An experimental structure.

**Figure 7.**
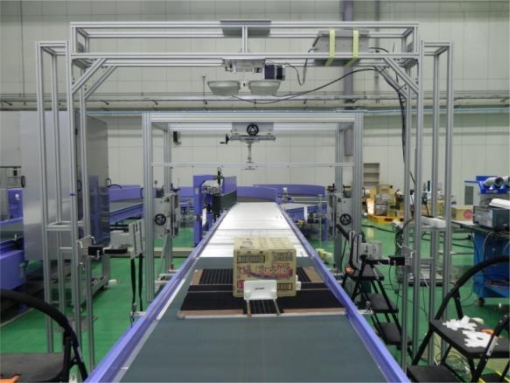
A picture of the experimental site with two antennas.

This section shows the result of experiments performed at the RFID/USN center recommended by EPCglobal in Song-do, Incheon, Korea. Figure 6 depicts the way in which the parts of an experiment are arranged, and Figure 7 shows an actual experimental site.

The experimental setup parameters are listed in Table 1. We have conducted two types of experiment with the conveyor belt gate system: employing an antenna control unit, and two arrayed fixed antennas. Linear and circularly polarized wave antennas were used for the experiments. For both the antenna control unit and fixed antenna experiments, we utilized two antennas using circular and linear type antennas.

**Figure 8.**
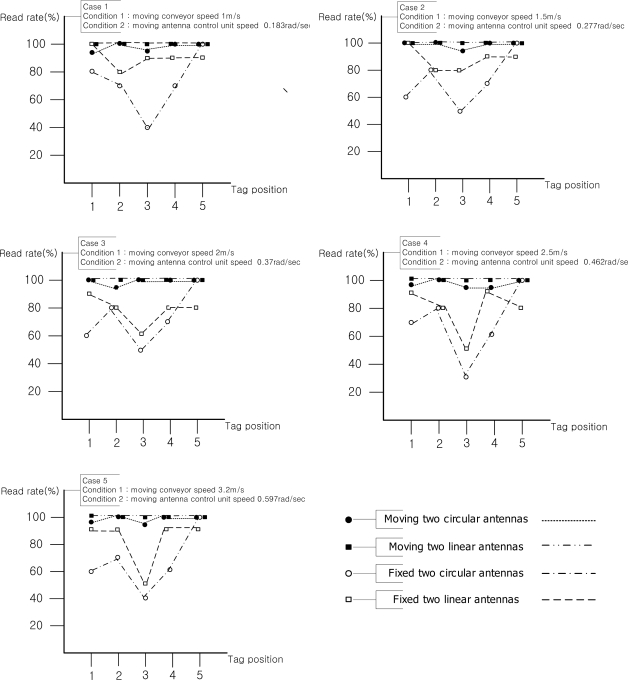
Direct comparison of recognition rates between moving and fixed antenna configurations.

Since tags isn’t attached to a regular face of a box in common material management systems using conveyor belts, the experiment was also carried out with tags attached on five faces of a box. In order to compare the recognition rates of each face of a box, each face was read 100 times. Recognition rates of each side of a box are shown in Figure 8.

In the experimental results, the fixed linearly polarized wave antenna worked best when the direction of tags and the beam pattern of the antenna of the RFID reader are aligned in a straight line. The recognition rates of the experiment on the front side (side 1) were high, regardless of the speed of the conveyor belt. The recognition rate characteristics between the RFID reader and RFID tag with the linear type antenna can be mainly affected by reading time. If a fixed type reader antenna has circular polarization characteristics, the recognition rate can be a little bit affected by the position of the attached tag. In the experimental results, when the antenna control unit has linear type reader antennas, the reading time was increased, since the polarization characteristic was matched with the attached RFID tag in the vertical direction. Therefore, 99% recognition rate was achieved regardless of the speed of the conveyor belt.

The tag on the rear face of a box has a bit lower average recognition rate (97%) than other sides of the box because it has the shortest reading time with a moving antenna unit. Although a tag on the rear face of a box was simultaneously ready to be read when the box started passing through the gate, tags on front, upper, left and right face of the box extensively received the beam pattern when an object was detected by the front photo sensor of the gate. Specifically, the tags except on the front are recognized by the antenna before and after passing the gate, and the front one can be read until the box reached the gate. However, the tag on the rear was ready for the antenna to read it after the box passed the gate and the tag information was only available until the box passed over the photo sensor located 1.5 meter backwards from the gate. In addition, the tag on the rear was affected by the scattering characteristics of moving antennas. Therefore, the tag the on rear face of a box has the shortest reading time and low recognition rates. From the experimental results above, we were able to find that the speed of conveyor belt, direction of tags attached, polarization characteristics and reading time of antenna on the antenna control unit are the contributing factors to improve overall recognition rates.

### 4.2. Linear Antenna *vs.* Circular Antenna

The experiments for this section were also conducted in the RFID/USN center guaranteed by EPCglobal. Three antennas was used for the experiments, since the conveyor belt gate system in the experimental site was designed to employ only three antennas on the left, top, right side on the gate.

**Figure 9.**
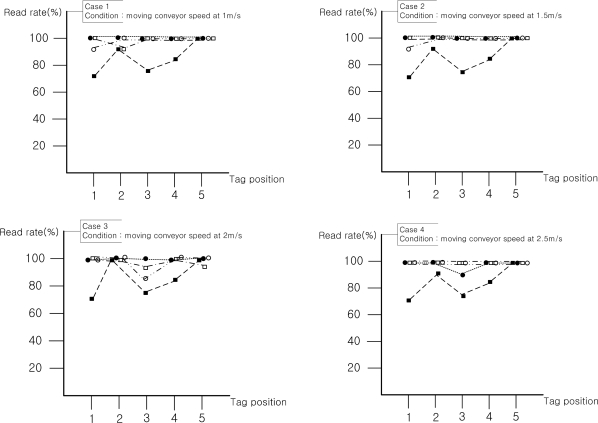

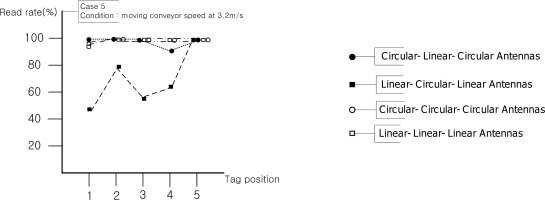
The experimental results of four different case studies using two types of antennas.

The experimental results are shown in Figure 9. Since the proposed experiment in this paper used two types of antennas, which were circularly and linearly polarized wave antennas, to verify polarization characteristics, we allocated the two types of antennas to three positions (left, top, and right of the gate) and were able to find that the circularly polarized antenna was the most suitable reader antenna in the general experiment setup. The linear and circular type antennas used have similar recognition rates according to the commercial velocity of the conveyor belt system. In these experiments the attachment position of the RFID tag is fixed in the vertical direction, but in the actual sites RFID tags can be attached on various directions. Therefore, the circularly polarized antenna is the most suitable reader antenna in the general experimental setup.

The experimental results show that the recognition rates of the system are significantly affected by the antenna polarization characteristics and speed of the conveyor belt. Since the recognition rates of the experiments using fixed two antennas cannot satisfy the recommendationx of EPCglobal, the structure is available for commercial systems. The experimental results of three antennas from LLL and CCC configurations at the conveyor belt speeds of 2.5 and 3.2 m/s represent 99% recognition rates, and total recognition rates of 99%, which is enough to commercialize the system, were shown in some experiments of the proposed antenna control unit with the LL structure at the conveyor belt speeds of 2.5 and 3.2 m/s, so we can say that the proposed antenna control unit meets the recognition conditions of EPCglobal although it has fewer antennas and smaller portal gate size than the conventional RFID conveyor belt gate system.

In terms of recognition rate, which is the most important factor in the RFID system, the proposed antenna control unit can be an excellent system to apply to various sites solving several problems such as polarization characteristics of reader antenna and tag locations, size of the gate, and distance between tags and reader. However, miniaturization and stabilization of the antenna control unit will be required through further experiments and research.

## 5. Conclusions

This paper presents a RFID conveyor belt gate system with a novel concept, utilizing an antenna control unit. In order to improve the recognition rate of the antenna, we have arranged two commercial antennas in the same row instead of designing an array antenna. Controlling the antenna to move along with materials has provided longer reading times to the system. The experimental results with the proposed novel structure show recognition rates of 97–99% which are the same as or better than the results of commercial structures employing multiple antennas and fixed structure of the same configuration. The proposed system has the potential to open a new regime for the design and use of RFID conveyor belt systems.

## Figures and Tables

**Table 1.** Experiment setup (EPCglobal standard setup).

**Conditions**	**Specifications**
**Speed of antenna control unit**	0.183, 0.277, 0.37, 0.462, 0.597 rad/s
**Speed of conveyor belt speed**	1, 1.5, 2, 2.5, 3.2 m/s
**RFID reader**	ALR-9800/output power of 20 dBm
**RFID tag**	Squiggle tag (70 mm × 19 mm)/Linear type antenna
**Gate height from conveyor belt**	1.25 m
**Humidity**	40%
**Temperature**	25 °C
**Experiment count**	100 iteration
**Size of the box that attached tags**	530 × 525 × 472 mm

**The position of RFID tags** **1, 2, 3, 4, 5**	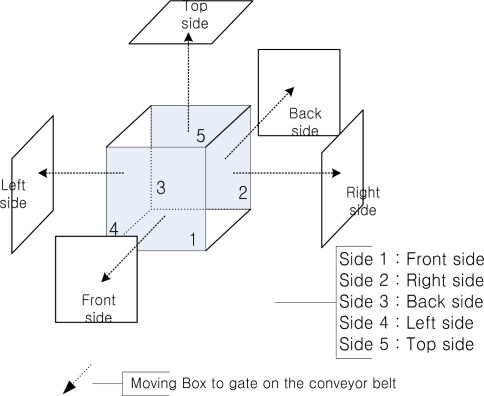

## References

[1] Weinstein R (2005). RFID: A technical overview and its application to the enterprise. IT Prof.

[2] Medeiros CR, Costa JR, Fernandes CA UHF RFID smart conveyor belt with confined detectionrange.

[3] Xing W, Dong W Experiment study on RFID performance factors of conveyor belt system using DOE methodology.

[4] (2006). Dynamic Test: Conveyor Portal Test Methodology.

[5] Xie L, Sheng B, Tan CC, Han H, Li Q, Chen D Efficient tag identification in mobile RFID systems.

[6] Kabadurmus O, Kilinc S, Behret H, Uygun G Performance evaluation of operational parameters on RFID controlled conveyor system.

[7] Weinstein R (2005). A technical overview and its application to the enterprise. IT Prof.

[8] Roberti M Case Study: Wal-Mart’s Race for RFIDi.

[9] Iwasaki H, Nakajima T, Suzuki Y (1995). Circularly polarized self-diplexing array antenna with higher gain operation. IEEE Tans. Antenn. Propag.

[10] Mailloux RJ, McIlvenna JF, Kernweis NP (1981). Microstrip array technology. IEEE Trans Antenn Propag.

[11] Munson RE (1974). Conformal microstrip antennas and microstrip phase arrays. IEEE Trans Antenn Propag.

[12] Orecchini G, Li Y, Tentzeris MM, Roselli L High directivity passive UHF RFID tag with dual-radiating-body antenna.

[13] Aryal G, Mapa L, Camsarapalli SK Effect of variables and their interactions on RFID tag readability on a conveyor belt-factorial analysis approach.

[14] Huang C-T, Lo L-W, Wang W-L, Chen H-L A study for optimizing the reading rate of RFID tagged cartons in palletizing process.

[15] Michael G, Markus B Experimental evaluation of RFID gate concepts.

